# MET: a Java package for fast molecule equivalence testing

**DOI:** 10.1186/s13321-020-00480-1

**Published:** 2020-12-17

**Authors:** Jördis-Ann Schüler, Steffen Rechner, Matthias Müller-Hannemann

**Affiliations:** Institute of Computer Science, Martin Luther University Halle-Wittenberg, Von-Seckendorff-Platz 1, 06120 Halle, Germany

**Keywords:** Molecule isomorphism, Molecule equivalence, Molecular graph

## Abstract

An important task in cheminformatics is to test whether two molecules are equivalent with respect to their 2D structure. Mathematically, this amounts to solving the graph isomorphism problem for labelled graphs. In this paper, we present an approach which exploits chemical properties and the local neighbourhood of atoms to define highly distinctive node labels. These characteristic labels are the key for clever partitioning molecules into molecule equivalence classes and an effective equivalence test. Based on extensive computational experiments, we show that our algorithm is significantly faster than existing implementations within SMSD, CDK and RDKit. We provide our Java implementation as an easy-to-use, open-source package (via GitHub) which is compatible with CDK. It fully supports the distinction of different isotopes and molecules with radicals.

## Background

**Fig. 1:**
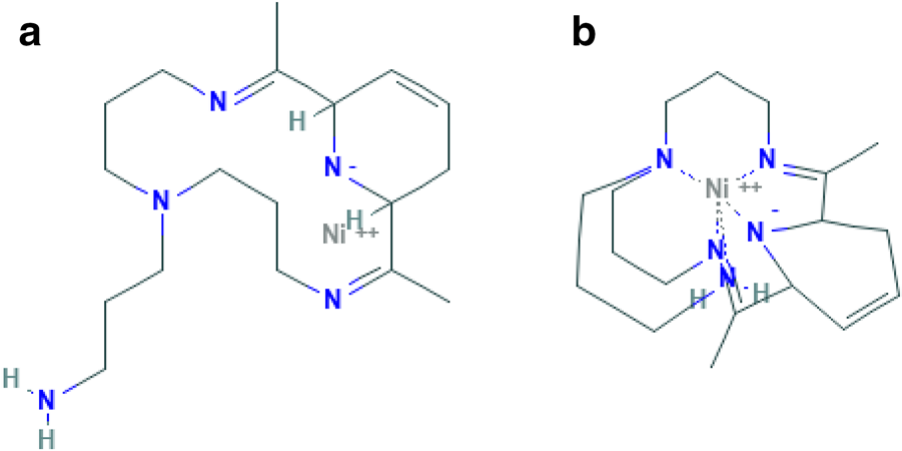
Molecule **a** is the image from the PubChem database for the molecule with the CID 50934715. Molecule **b** is the image from the PubChem database for the molecule with the CID 6397461. Both molecules are equivalent in 2D

The analysis of molecules is an important part of cheminformatics. As atoms and bonds can be combined in a multitude of ways, a huge number of different molecules can be formed. Databases like PubChem, KEGG, or ChEBI store millions of molecules. Querying whether some molecule is included in such a database requires to test whether this molecule is equivalent to some existing one, i.e. whether they share the same structural and chemical properties. For example, it is not at all obvious that the two molecules (PubChem CID: 50934716 and 6397461), visualized in Fig. 1, are equivalent in 2D (not regarding stereochemistry). In this paper, we study the problem of testing whether two molecules are equivalent.

In an abstract mathematical way, molecules can be represented as graphs with atoms as nodes and bonds as edges. Chemical properties of atoms can be modelled as integral or real-valued node labels. To test whether two molecules are equivalent thus means to test whether the two associated labelled graphs are isomorphic.

The general graph isomorphism problem is neither known to be in P nor to be NP-complete [1, 2]. In a recent breakthrough paper, Babai presented a quasipolyonomial-time algorithm for the graph isomorphism problem in general graphs [3]. This algorithm runs in time $O(n^{\text {polylog}(n)})$ where *n* is the number of nodes and polylog(*n*) is some polynomial in $\log (n)$. For many special cases, stronger results are known. For example, for planar graphs, the problem can be solved in polynomial time [4]. A lot of molecules like DNA, RNA, or fullerenes can be represented as planar graphs. However, it is known that other molecule classes like inorganics or linked polymer networks cannot [2, 5]. In pioneering work, Luks presented a polynomial-time isomorphism algorithm for graphs of bounded degree [6]. Since bond and atom types are bounded, this immediately implies the polynomial-time solvability of molecular equivalence via standard transformations from molecular, labelled graphs to simple graphs [2]. The corresponding polynomials are, however, of high degree so that algorithms require different techniques to be usable in practice.

There are many algorithms to test whether two labelled graphs are isomorphic. One possibility is to first transform molecule graphs into simple graphs without node labels, and then to solve the classical graph isomorphism problem [7]. In contrast, many other approaches, like ours, work directly on labelled graphs. McKay’s famous nauty algorithm [8] is based on the idea of finding a canonical form of a graph *G*, i.e. a labelled graph *C*(*G*) that is isomorphic to *G*, often referred to as canonical labelling, such that every graph that is isomorphic to *G* has the same canonical form. Then testing whether two graphs are isomorphic reduces to merely checking equality of their corresponding canonically labelled graphs, which is easy. The hard part is to compute the canonical labellings. The nauty package is freely available; although known to have exponential runtime on some inputs, it performs very well in practice. The probably first practically usable algorithm goes back to Ullmann [9], who used recursive backtracking to solve the isomorphism problem (actually, his approach even solves the NP-complete subgraph isomorphism problem). A substantially improved version appeared in [10]. The general idea of iteratively extending a partial solution using certain feasibility criteria has been employed in several variants of VF/VF2/VF2 Plus/VF2++ algorithm [4, 11–13]. Experimental studies showed that the VF2++ algorithm is very efficient in practice [4]. It seems to be the fastest available code up to now.

**Fig. 2:**
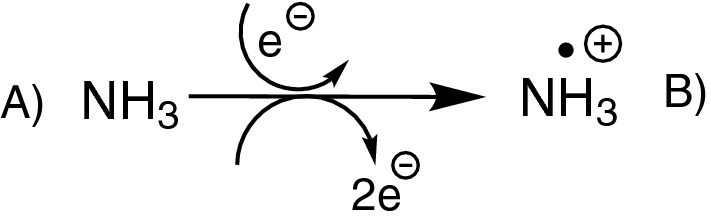
Example: ionisation of ammonia by electron impact reaction. These two molecules are recognized as equivalent by the isomorphism test of CDK

**Fig. 3:**
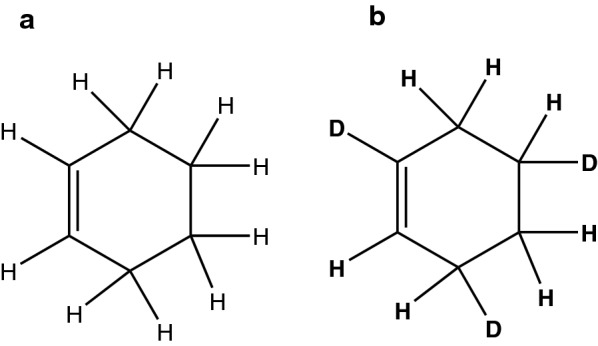
Structure **a** cyclohex-1-ene, structure **b** cyclohex-1-ene with three replaced hydrogens by deuterium. These two molecules are recognized as equivalent by SMSD

In the context of cheminformatics, algorithms for testing molecule equivalence are implemented in widely-used software packages like RDKit [14], CDK [15] and SMSD [16]. These tools work well with a multitude of molecules. However, they do not always respect all chemical properties, like deuterium and radicals (see Figs. 2, 3). Other algorithms implemented in CDK and SMSD do respect these properties but suffer from large running times.

Many scientists in cheminformatics use canonical Simplified Molecular Input Line Entry Specification (SMILES) [17, 18] or InChI (International Chemical Identifier) [19, 20] to represent molecules as strings [21, 22]. To test whether two molecules are equivalent, it suffices to check whether the associated strings are identical. To create such strings, a canonical labelling problem has to be solved. SMILES or InChI strings can be created by CDK or RDKit [15, 21]. Several limitations of the SMILES format exist, most importantly, that there is no standard way to generate a canonical representation [22]. We will later compare these approaches with ours.

*Contribution* To improve the current situation, we developed and implemented an algorithm for testing molecule equivalence in 2D. Our software is called MET (Molecule Equivalence Tester) and is available at https://www.github.com/jaschueler/MET/. Our software is designed and has been engineered to

1. consider chemical properties like deuterium and radicals in the equivalence test,
2. be highly competitive with the isomorphism algorithms from CDK and SMSD as well as with established SMILES and InChI methods, and
3. be compatible with CDK such that it can be easily integrated into existing CDK applications.

For testing molecular equivalence, our key contribution is to define highly distinctive node labels encoding both chemical properties and the local structural neighbourhood of an atom up to a certain depth. In order to achieve excellent performance, we have carefully engineered the appropriate choice of properties and the neighbourhood depth.

In the following section, we describe our method and the associated techniques. Afterwards, we experimentally evaluate our algorithm and show that it largely outperforms existing implementations from CDK, SMSD, and RDKit.

## Methods

Let us start by formally defining the equivalence of molecules.

### Preliminaries: molecule equivalence

In our application, we model a molecule as a graph $G = (V, E)$ with node set *V* and edge set *E*. Each node *v* has an associated node label $\ell (v) \in {\mathbb {R}}^s$ of *s* (integral or real) numbers, each of which represents a certain atom property. A very simple choice of node labels would be, for example, to simply use the atomic number of the corresponding atom (in which case *s* would be one). Later, we will explain in detail how more sophisticated chemical and structural properties can be encoded into labels. We define that two molecules $G_1 = (V_1, E_1)$ and $G_2 = (V_2, E_2)$ are equivalent if and only if there is an isomorphism *f* between $G_1$ and $G_2$ i.e. a bijective function $f :V_1 \rightarrow V_2$ such that

- $\{ f(u), f(v) \} \in E_2$ if and only if $\{u,v\} \in E_1$, and
- $\ell _1(v) = \ell _2(f(v))$ for each node $v\in V_1$.

The first condition ensures that both graphs have the same structure while the second guarantees that also the node labels are compatible.

### High-level description

Our algorithmic approach works as follows. The algorithm gets as input two molecules. For simplicity, we assume that these are given by their CDK representations. Alternatively, one may pass these molecules by some standard file description, for example, in SDF format. Our algorithm consists of two phases.

1. In the first phase, the algorithm converts both molecules into labelled graphs. In doing so, each molecule is transformed into a graph $G=(V,E)$ with node set *V* and edge set *E*. For each atom, we introduce a node $v \in V$ and for each bond, we introduce an edge $e \in E$. In doing so, we create two graphs $G_1 = (V_1, E_1)$ and $G_2 = (V_2, E_2)$. In the second step, we create node labels $\ell _1 :V_1 \rightarrow {\mathbb {R}}^s$ and $\ell _2 :V_2 \rightarrow {\mathbb {R}}^s$. It is crucial that two nodes gain identical labels if and only if their associated atoms have identical properties. Our key contribution is to compute highly distinctive node labels.
2. In the second phase, we first run quick pre-test to check whether $G_1$ and $G_2$ are certainly not isomorphic. If this pre-test does not preclude the isomorphism, we use an isomorphism algorithm to decide whether the labelled graphs are isomorphic.

In the following, we give a detailed description of both phases.

### Phase one

In the first phase, we transform the CDK molecule representations (regardless of stereochemistry) to labelled graphs. For this purpose, we replace each atom by a node and each bond by an edge. To conserve the chemical properties of each atom, each node gets a label, on which we will focus next.

#### Atom properties

Our software is designed to let the user choose the chemical properties that should be respected during the equivalence test. In our applications, we consider the following atom properties:

- atomic number
- count of hydrogens and deuterium
- formal charge
- count of single electrons (radicals)
- count of single bonds, double bonds and triple bonds

Additional properties can easily be integrated into our algorithm, as long as they can be represented as real or integral numbers. For example, chemical properties like radius or partial charges can be represented as real numbers and might be helpful, if available.

**Fig. 4:**
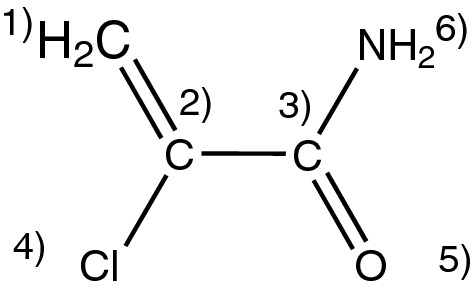
Molecule 2-chloroprop-1-enamide for the explanation of the classical atom properties

Table 1 shows the atom properties of the example molecule in Fig. 4.

**Table 1:** list of classical atom properties for the molecule in Fig. 4

	Atom 1	Atom 2	Atom 3	Atom 4	Atom 5	Atom 6
Atomic number	6	6	6	17	8	7
Count of hydrogens	2	0	0	0	0	2
Count of deuterium	0	0	0	0	0	0
Formal charge	0	0	0	0	0	0
Count of single electrons	0	0	0	0	0	0
Count of single bonds	0	2	2	1	0	1
Count of double bonds	1	1	1	0	1	0
Count of triple bond	0	0	0	0	0	0

Based on the selected properties, each node *v* gets an associated label $\ell (v) = (p_1, p_2, \ldots , p_s)$, where each $p_i$ represents a certain atom property. It is crucial that two nodes gain identical labels if and only if their associated atoms have identical properties.

#### Neighbourhood descriptors

In addition to the chemical properties, each node *v* gets an additional structural property *d*(*v*) called neighbourhood descriptor. This is a (real or integral) number that encodes information on the local neighbourhood of this node. We intend to give nodes identical neighbourhood descriptors if

- they have the same set of chemical properties, and
- their local neighbourhood is identical.

To compute the neighbourhood descriptors, we iteratively define for each node *v* an integer $d_i(v)$ that characterizes its local neighbourhood up to a depth of *i*. Initially, we define

$$\begin{aligned} d_0(v) := hash (\ell (v)). \end{aligned}$$

Here, $hash$ denotes a generic hash function that maps tuples of (real or integral) numbers to integers. Consequently, the initial descriptor $d_0(v)$ contains information only on each node itself. For $1 \le i \le k$ (where the maximum depth *k* is a parameter on which we focus soon), we define

$$\begin{aligned} d_{i}(v) := \sum _{(v,w) \in E} d_{i-1}(w). \end{aligned}$$

Having calculated $d_i(v)$ for $0 \le i \le k$, we use

$$\begin{aligned} d(v) := hash (d_0(v), d_1(v), \ldots , d_k(v)) \end{aligned}$$

as the neighbourhood descriptor of the node *v*.

**Fig. 5:**
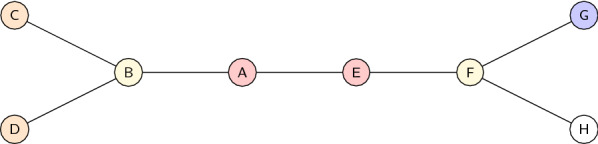
Example graph. The nodes *A* and *E*, *C* and *D*, as well as *B* and *F* are supposed to have identical chemical properties and marked with the same colours

Table 2 demonstrates the calculation of the neighbourhood descriptors on the example shown in Fig. 5.

**Table 2:** Example of the calculation of the neighbourhood descriptor based on Fig. 5

	$d_0$	$d_1$	$d_2$	$d_3$
A	5	8	27	39
B	3	19	14	65
C	7	3	19	14
D	7	3	19	14
E	5	8	25	41
F	3	17	14	59
G	10	3	17	14
H	2	3	17	14

The values in $d_0$ are self-chosen and do not follow a special semantic

We briefly point to some instructive observations.

- As the nodes *C* and *D* are supposed to have identical chemical properties, and they additionally have the same neighbourhood structure, they gain identical values of $d_i$ for all $i \ge 0$. Consequently, they gain the same neighbourhood descriptor *d* (not shown in the example).
- The nodes *G* and *H* are supposed to have different chemical properties (thus $d_0(G) \not = d_0(H)$) but their neighbourhood structure is exactly symmetric (thus $d_i(G) = d_i(H)$ for all $i \ge 1$). Nevertheless, they will get different neighbourhood descriptors.
- The nodes *A* and *E* have identical chemical properties (thus $d_0(A) = d_0(E)$). Furthermore, their direct neighbourhood is symmetric (thus $d_1(A) = d_1(E)$). However, their local neighbourhood differs when considering a depth of two or more.

*Maximum depth*

It is an important question of how to choose the maximum neighbourhood depth *k*. On the one hand, a large value of *k* will include more information on the local neighbourhood of each node, and will thus potentially decrease the running time of the isomorphism algorithm. On the other hand, calculating the neighbourhood descriptors for each node of a molecule graph $G=(V,E)$ requires a running time of $O(k \cdot |E|)$. Thus, we might want to choose *k* as a small constant. Later, in the experimental results section, we will study the influence of *k* on the running time in a benchmark experiment.

### Phase two

In the second phase, we test whether two labelled graphs $G_1$ and $G_2$ with node labels $\ell _1$ and $\ell _2$ are isomorphic.

*Pre-test*

Before starting the actual isomorphism test, we run a fast pre-test to quickly detect whether the graphs are certainly not isomorphic. For this purpose, we compare the number of atoms, bonds, hydrogens, deuterium, formal charge and single electrons of both molecules, which all must be equal. In addition, we construct the label sets $\{ \ell (v) : v \in V_1 \}$ and $\{ \ell (v) : v \in V_2 \}$ and test whether both sets are identical. If not, we can be sure that the two molecules are not equivalent.

*Equivalence test* After the pre-test, we try to find an isomorphism between $G_1$ and $G_2$. In this paper, we tested two algorithms:

- First, we tested the $\underline{\texttt {VF2++}}$ algorithm included in the Lemon library [13, 23]. Recent work showed that this algorithm is very fast on large instances [4]. However, this algorithm is implemented in C++. This is a small disadvantage for native Java application, like our implementation, as calling this algorithm requires system calls and thus reduces the portability of the Java code.
- Second, we tested our own implementation of a generic backtracking algorithm, which we will describe in a moment. In contrast to VF2++, this algorithm is implemented in native Java and is thus more portable. As a drawback, our algorithm has been less engineered and might therefore be less efficient than VF2++.

We now give a rough description of our isomorphism algorithm. The algorithm gains as input two graphs $G_1$ and $G_2$ with associated node labels $\ell _1$ and $\ell _2$.

*Candidate sets*

For each node $v \in V_1$, the algorithm maintains a candidate set

$$\begin{aligned} can_1 (v) := \{ w \in V_2 :\ell _1(v) = \ell _2(w) \} \end{aligned}$$

of nodes in $G_2$ that may be assigned to the node *v*. Vice versa, the algorithm maintains for each node $w \in V_2$ in $G_2$ the candidate set

$$\begin{aligned} can_2 (w) := \{ v \in V_1 :\ell _1(v) = \ell _2(w) \} \end{aligned}$$

of nodes in $G_1$ that may be assigned to *w*.

*Backtracking*

The heart of the isomorphism algorithm is the following backtracking algorithm (see Algorithm 1).

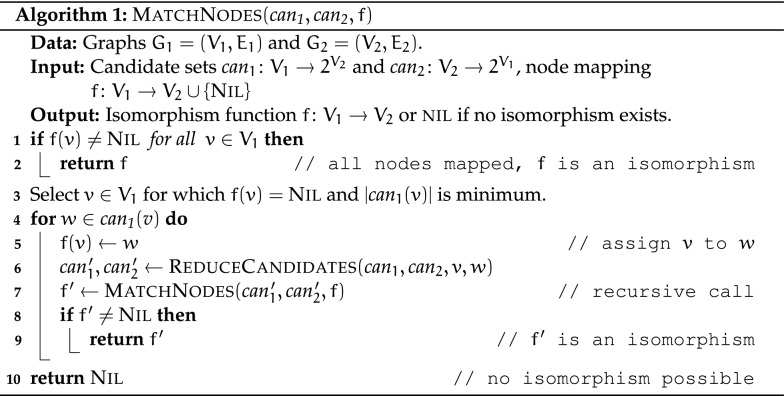

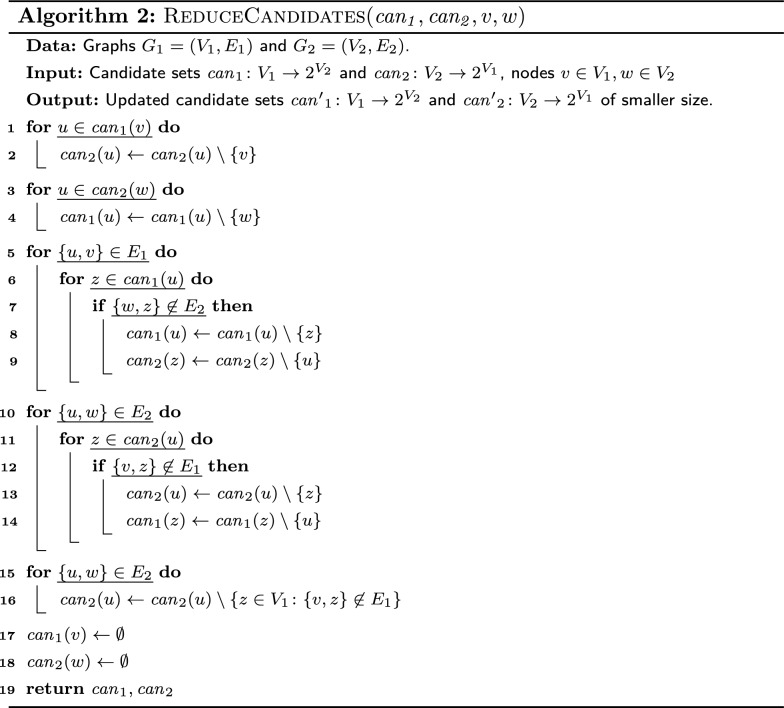





 The algorithm selects a node $v \in V_1$ with a minimum-sized candidate set and tries to assign *v* to one of its candidate nodes, say to $w \in can_1(v)$. By assigning *v* to *w*, we may reduce several candidate sets (see Algorithm 2):

- Obviously, *v* and *w* can be removed from every candidate set in which they were included so far.
- For each edge $\{u,v\} \in E_1$, there must be an edge $\{z, w\} \in E_2$. Thus, for each edge $\{u,v\} \in E_1$, we can remove from $can _1(u)$ each node that is not adjacent to *w*.
- Symmetrically, we can remove from $can _2(z)$ each node that is not adjacent to *v*.
- Furthermore, the candidate sets of *v* and *w* can be cleared.

In doing so, we reduce the candidate sets of the nodes. (Note that candidate sets will only be reduced, never increased by the assignment of two nodes.)

*Recursion*

After assigning two nodes to each other, we recursively try to find an assignment to the remaining nodes. This may or may not be successful.

- If we successfully assigned each node from $G_1$ to some node from $G_2$, we successfully determine that both graphs are isomorphic. We return with a positive answer and the isomorphism function *f*.
- If we did not succeed in recursively assigning the remaining nodes, we undo our latest modifications on the candidate lists, backtrack and try to assign to *v* the next node from its candidate set. After all candidate nodes have been tested this way (and we did not return with a positive answer), we know that $G_1$ and $G_2$ cannot be isomorphic.

Note that the isomorphism $f :V_1 \rightarrow V_2$ is easily obtained by this procedure.

The efficiency of our algorithm highly depends on the sizes of the candidate sets. Recall that, the larger the maximum depth *k*, the smaller the candidate sets will be, as the node labels become more distinctive.

## Experiments, results, and discussion

To quantify the performance of our implementation in comparison to existing methods, we designed a benchmark experiment.

*Benchmark experiment*

In each of the following experiments, we partitioned a certain SDF file with molecules into its equivalence classes, i.e. into maximum sized subsets of molecules in which all molecules are pairwise equivalent. Each equivalence class has a designated member called it‘s representative. Our general process is sketched as follows.

1. We read one molecule after the other from the input file and construct the associated graph $G=(V,E)$ with node labels $\ell$.
2. To insert *G* into its equivalence class, we use the property set $\{ \ell (v) : v \in V) \}$ to compile a list of representatives that have the same property set and thus maybe equivalent to *G*. Let $L_G$ denote this list.
3. For each representative $R \in L_G$, we test whether *G* is equivalent to *R*. To this end, we use one of the following algorithms/approaches:
  MET: using the isomorphism algorithm described in the Methods section,
  VF2++: using the VF2++ algorithm from the Lemon library [4],
  CDKMCS, MCSPlus, Vlib, Default from the SMSD package [16],
  CDK: SMILES: using CDK to create canonical SMILES [15],
  RDKit: SMILES: using RDKit to create canonical SMILES [21],
  CDK: InChI: using CDK to create an InChI representation [15].
  If *G* and *R* are found to be equivalent, we add *G* to *R*’s equivalence class and continue with the next molecule. Note that each of the algorithms may give a different result.
4. If *G* is equivalent to none representative in $L_G$, we create a new equivalence class represented by *G*.

*Experimental environment* All experiments were conducted on a Debian GNU/Linux 10 system with 38 CPU cores and 250 GB main memory. We used Java on version 12, CDK 2.3, RDKit 2018.09.1 (Ubuntu package) and SMSD 2.2.0.

### Parameter tuning and optimization

In the first four experiments, we tried to find an optimum value of the neighbourhood depth parameter *k*. In addition, we evaluated which graph isomorphism algorithm works best in the second phase of our algorithm.

**Fig. 6:**
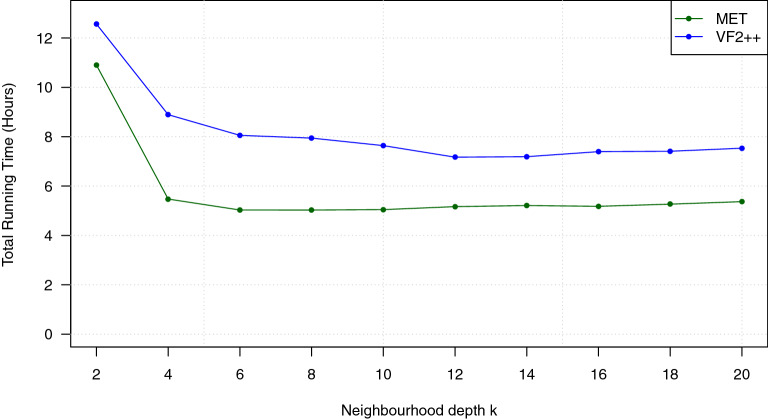
Influence of the neighbourhood depth parameter *k* on the running time of the algorithms MET and VF2++

*Experiment 1* In a first experiment, we studied the influence of the neighbourhood depth parameter *k* on the running time of the MET and VF2++ algorithms. For this purpose, we ran our benchmark experiment for several values of *k* and measured the associated total running time. As the input data set, we used the set of 95 945 527 molecules that are listed in the PubChem database (October 2019), excluding molecules that solely consist of hydrogen or deuterium. Figure 6 shows the result of this experiment. We summarize some essential observations.

- We observe that a very small value of *k* induces a large running time. This is not surprising, as, with small *k*, the node descriptors carry little information on the local neighbourhood of each atom. The total running time reaches a minimum at $k=6$.
- Increasing the value of *k* beyond 6 does not significantly decrease the running time. Instead, the running time stays nearly constant for $k \ge 6$. We conclude that a value of $k=6$ might be a reasonable value for our type of application.
  Our observations agree with the results from related experiments on molecular fingerprints for small organic molecules [24, 25], where it has been found that a diameter of 4 or 6 gives the best performance.
- We further observe that the MET algorithm slightly outperforms VF2++ for all values of *k*. This may have several reasons. For example, since VF2++ is implemented in C++, we need to call it using the Java Native Interface, which induces additional overhead. In any case, we conclude that MET is slightly superior to VF2++.

In further experiments, we use $k=6$ as the maximum neighbourhood depth.

*Experiment 2* Next, we further studied in more detail whether MET or VF2++ work best in the second phase of the equivalence test. In contrast to the previous experiment, we now consider data sets of different molecule sizes. For this purpose, we partitioned the PubChem data set into seven groups of molecules of approximately similar size.

- 56,410,359 molecules with up to 25 atoms,
- 35,528,631 molecules with 26 to 45 atoms,
- 3,773,370 molecules with 46 to 100 atoms,
- 168,714 molecules with 101 to 150 atoms,
- 40,251 molecules with 151 to 200 atoms,
- 22,745 molecules with 201 to 400 atoms, and
- 1457 molecules with more than 400 atoms.

**Fig. 7:**
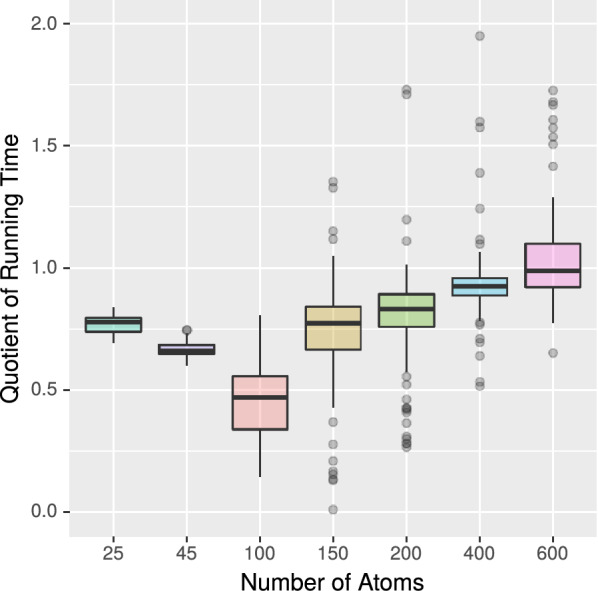
Running time of MET divided by running time of VF2++ (with $k = 6$). Medians are marked as bold horizontal lines

We ran our benchmark experiment for each group individually and measured the associated running times for MET and VF2++. By calculating the quotient of both running times, we quantify the speed-up of one algorithm to the other. A quotient of one means that both algorithms are equally fast, a quotient of less than one means that MET is faster than VF2++. Figure 7 shows the result of this experiment.

- We observe that for each group, MET is superior to VF2++ in the majority of cases (as the medians lie below one). However, there are outliers in both directions.
- While MET is superior for the majority of small molecules, it becomes less efficient when the number of atoms grows larger.

We conclude that our isomorphism algorithm is very well suited for molecules with up to 400 atoms, while VF2++ is comparably efficient but suffers from additional overhead.

**Fig. 8:**
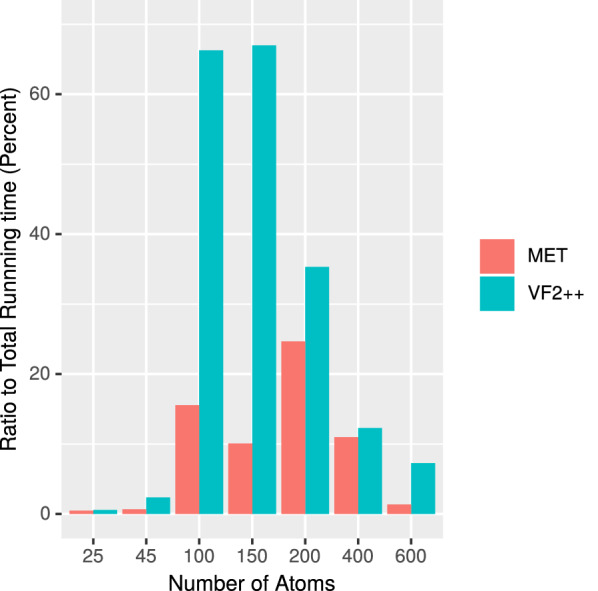
Ratio of the isomorphism algorithms of MET and VF2++ (phase 2) on the total running time in percent for the groups, with $k=6$

*Experiment 3* Based on Experiment 2 we next considered the ratio between the running time of the equivalence test and the total running time. Therefore, we use the data set from Experiment 2 and individually measure the running time of the second phase only. Dividing this time through the total running time gives the ratio of the isomorphism algorithm (plus pre-test) on the total running time. In Fig. 8 we show the result of our experiment.

- We observe that the ratio of the isomorphism algorithm of MET is smaller than the algorithm of VF2++ for all molecule groups.
- Especially, for the molecules with 50–200 atoms the algorithm of MET is significantly faster.

This result underlines the result of Experiment 2 and thus the suggestive usage of MET for molecules with up to 400 atoms.

*Experiment 4* In the previous experiment, we tested a lot of molecule pairs which are not equivalent. Most equivalence test failed during our pre-tests. In this experiment, we studied the efficiency of MET and VF2++ algorithms on a data set for which we a priori know that all molecules are equivalent.

**Fig. 9:**
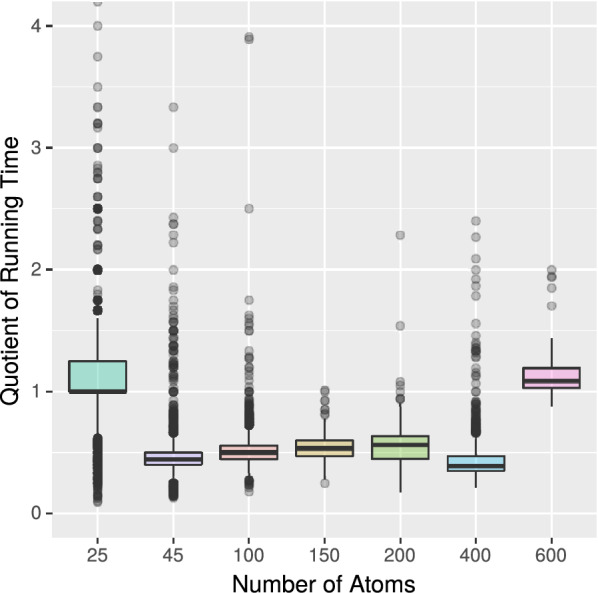
Running time of MET divided by running time of VF2++ (with $k = 6$) on a data set consisting only of isomers. Medians are marked as bold horizontal lines

For each group of molecule sizes, we selected a subset of equivalence classes as input for our benchmark experiment. In doing so, we made sure that all pairs of tested molecules are equivalent. We measured the running time of our benchmark experiment on these input files. Figure 9 shows the result of this experiment.

- We observe that both algorithms work approximately equally well for very small and very large molecules.
- For medium-sized molecules MET is superior to VF2++.

### Performance comparision

In our final set of experiments, we evaluated how the MET algorithm compares to the established equivalence tests from SMSD and to the methods based on canonical SMILES and InChI from CDK and RDKit.

*Experiment 5*

As it will become apparent from our experiment, the running time of some equivalence algorithms is very large. Consequently, we did not run all algorithms on the complete data set. In contrast, we just partitioned a small subset (23,254 molecules) of the PubChem database into its equivalence classes and measured the associated running time. Table 3 shows the running times for this process.

**Table 3:** Running time and number of deviating results for a small set of molecules (using a neighbourhood depth of $k=6$)

Algorithm	Milliseconds	Deviating results
MET	7693	0/692
VF2++	6488	0/692
SMSD: CDKMCS	18,677	9/692
SMSD: MCSPlus	2,740,136	9/692
SMSD: Vlib	4,940,974	0/692
SMSD: Default	24,902 968	0/692
RDKit: SMILES	54,872	0/692
CDK: SMILES	7399	0/692
CDK: InChI	6 563	0/692

- First, we observe that two algorithms (CDKMCS, MCSPlus) from the SMSD do not correctly identify all pairs of equivalent molecules. For example, the structures of methadone hydrochloride (PubChem CID: 14184) and levomethadone hydrochloride (PubChem CID: 22266) in 2D representation are evaluated as being different. Interestingly, these molecules do not contain radicals or isotopes.
- Considering the running time, we observe that our approach outperforms all algorithms from SMSD by several orders of magnitude. Consequently, we exclude these algorithms from further experiments.
- We observe that in this small sample the equivalence tests based on canonical SMILES and InChI give correct results. We further observe that the running time of RDKit is noticeable larger than that of CDK.

From this experiment, we conclude that only the SMILES and InChI based algorithms from CDK are competitive with MET and VF2++. Thus, we will further examine those algorithms on the large data set.

**Fig. 10:**
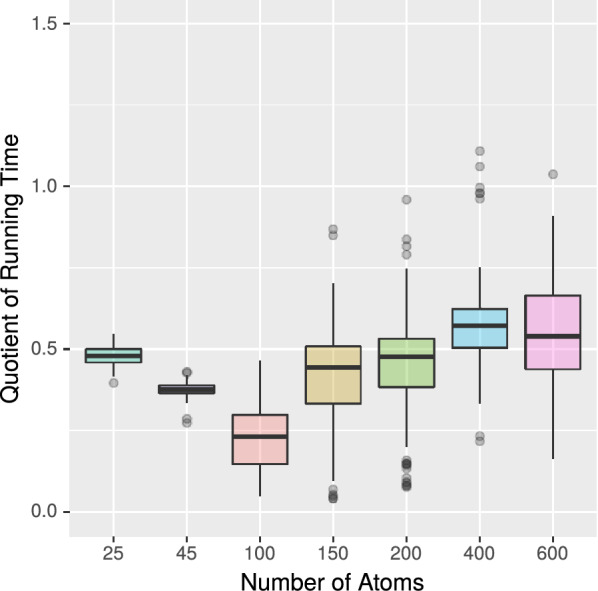
Running time of MET (with $k = 6$) divided by the running time of InChI (CDK). Medians are marked as bold horizontal lines

**Fig. 11:**
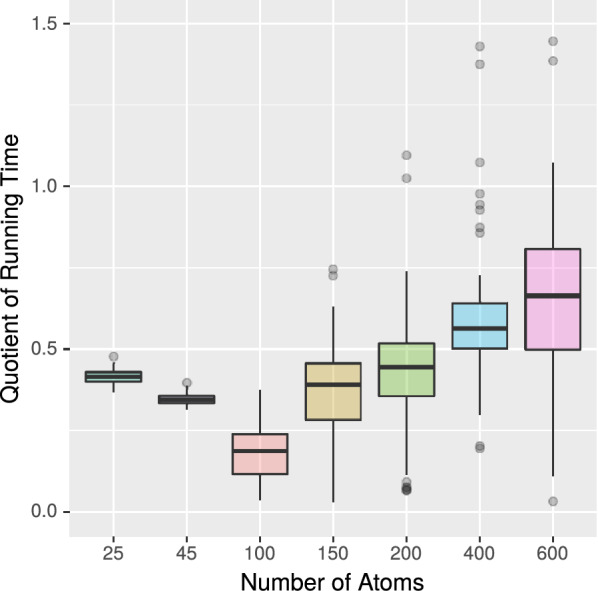
Running time of MET (with $k = 6$) divided by the running time of SMILES (CDK). Medians are marked as bold horizontal lines

*Experiment 6* In our final experiment, we compare the running time of the SMILES and InChI based CDK methods with that of MET and VF2++. For this purpose, we ran these algorithms on the large data set from Experiment 2. Table 4 and Figures 10 and 11 show the results.

**Table 4:** Running time and number of deviating results for the PubChem data set (using a neighbourhood depth of $k=6$)

Algorithm	Milliseconds	Deviating results
MET	18,206,705	0/18554268
VF2++	29,093,114	0/18554268
CDK: SMILES	63,182,381	1190/18554268
CDK: InChI	38,050,677	3245/18554268

- Considering CDK’s InChI method, we observe that MET outperforms CDK by a factor of about 2. In addition, we observe that the CDK method produce deviating results for 3245 molecule pairs, including 3 pairs for which at least one InChI could not be constructed by the CDK method. The remaining deviating results are false positives from CDK. For example, the molecules with CID 18671247 and 60160843 gain the same InChI although they differ in the position of some double bond. Another example is the molecules with CID 5151983 and 379953, which differ in the position of some proton but gain the same InChI.
- Considering the SMILES method, we observe that MET’s running time is just one third of CDK’s. There are 1190 out of 18554268 molecule pairs for which the SMILES method gives a different result than MET. These different results are in all cases false negatives from CDK. For example, CDK generates for the molecules with CID 414487 and 49791694 different SMILES. In comparison, the PubChem database and the canonical SMILES generation by RDKit show the same SMILES for these molecules. Included in the 1190 pairs are 93 for which CDK could not construct the associated SMILES.

From our final experiment, we conclude that MET is a significant improvement to existing tools as it does not depend on the error-prone construction of canonical SMILES or InChIs and is furthermore considerably faster than CDK and RDKit.

## Conclusion

In this article, we presented an algorithm for detecting the equivalence of molecules. Our algorithm exploits the chemical and structural properties of molecules to transform a molecule to a labelled graph. Our method is based on the construction of highly distinctive node labels that are used to decrease the running time of a isomorphism algorithm. Experimentally, we showed that it suffices to consider the local neighbourhood up to a depth of six. In its second phase, our algorithm uses a generic isomorphism algorithm for labelled graphs. Our experiments showed that our generic backtracking algorithm is competitive with the previously fastest implementation VF2++. In a set of experiments, we showed that our algorithm is faster than all algorithms currently implemented in SMSD, CDK, and RDKit. In addition, we found that our method is more robust than the methods included in CDK as it avoids the construction of SMILES or InChIs. As our software is compatible with CDK, it can easily be used to replace all current algorithms for equivalence testing from CDK or SMSD.

In the future, we plan to integrate our algorithm to the molecule fragmentation software ChemFrag [26]. Furthermore, we want to analyse the applicability of our algorithm for large molecules like proteins. In addition, we are going to consider the related problem whether some molecule is part of some larger molecule [27]. For this purpose, we need to solve the subgraph isomorphism problem, which is known to be NP-complete [1].

## Availability and requirements

- Project Name: Molecule Equivalence Tester (MET)
- Project home page: https://github.com/jaschueler/MET/
- Operating system(s): GNU/Linux.
- Programming language: Java 12
- Any restrictions to use by non-academics: None
